# A mechanism of glucose tolerance and stimulation of GH1 β-glucosidases

**DOI:** 10.1038/srep17296

**Published:** 2015-11-25

**Authors:** Yang Yang, Xinxin Zhang, Qiang Yin, Wei Fang, Zemin Fang, Xiaotang Wang, Xuecheng Zhang, Yazhong Xiao

**Affiliations:** 1 School of Life Sciences, Anhui University, Hefei, Anhui 230601, China; 2 Anhui Provincial Engineering Technology Research Center of Microorganisms and Biocatalysis, Hefei, Anhui 230601, China; 3 Department of Chemistry & Biochemistry, Florida International University, Miami, Florida 33199, United States

## Abstract

β-Glucosidases are enzymes that hydrolyze β-glycosidic bonds to release non-reducing terminal glucosyl residues from glycosides and oligosaccharides, and thus have significant application potential in industries. However, most β-glucosidases are feedback inhibited by the glucose product, which restricts their application. Remarkably, some β-glucosidases of the glycoside hydrolase (GH) 1 family are tolerant to or even stimulated by glucose. Elucidation of the mechanisms of glucose tolerance and stimulation of the GH1 β-glucosidases will be crucial to improve their application through enzyme engineering. In this study, by comparing the primary and tertiary structures of two GH1 β-glucosidases with distinct glucose dependence, some putative glucose-dependence relevant sites were mutated to investigate their exact roles. Both biochemical and structural characterization of the mutants suggested that some sites at the entrance and middle of the substrate channel regulate the effects of glucose, and the relative binding affinity/preference of these sites to glucose modulates the glucose dependence. A mechanism was therefore proposed to interpret the glucose dependence of GH1 β-glucosidases. This research provides fresh insight into our current understanding of the properties and mechanisms of GH1 β-glycosidases and related enzymes that modulate their activity via feedback control mechanism.

β-Glucosidases (EC 3.2.1.21) are a class of enzymes that hydrolyze terminal, non-reducing β-D-glucosyl residues with release of β-D-glucose^1^. β-glucosidases have potential applications in various industrial processes including the release of flavor components from flavor additives^2^,^3^ and the synthesis of glucosylated compounds such as ginsenoside^4^. Recently, β-glucosidases have drawn intensive attention because of their critical role in the biological conversion of cellulose to glucose which can be further utilized by yeast and other organisms to produce ethanol or other fuel products^5^,^6^,^7^,^8^,^9^. However, the application of β-glucosidase has been severely thwarted due to product inhibition of the enzyme^7^,^10^. Most β-glucosidases are sensitive to glucose^8^,^11^,^12^, whereas some β-glucosidases are tolerant to glucose^8^,^13^,^14^,^15^. Moreover, glucose tolerance in β-glucosidases is sometimes coupled with a stimulatory effect of the carbohydrate, which seems to be exclusive to some glycoside hydrolase (GH) family 1 β-glucosidases^16^,^17^,^18^,^19^. In biotechnology industries, application of β-glucosidases with glucose tolerance and stimulation can increase the efficiency of substrate degradation and reduce the cost of production^9^,^20^,^21^,^22^. Therefore, glucose-tolerant β-glucosidases have been attracting increasing attention in recent years^8^,^15^,^17^,^18^,^19^,^23^,^24^.

To date, the molecular basis of glucose tolerance and stimulation of β-glucosidases remains unclear. Several studies have suggested that these properties are regulated through an allosteric mechanism by glucose binding to other sites rather than the active site^15^,^23^. It has also been demonstrated that kinetic modulation of turnover number of these enzymes is associated with transglycosylation^17^,^18^. However, specific glucose-binding sites and modulation details, such as the mechanism of transglycosylation, have not been elucidated. A recent study on the structural bases for the glucose dependence of GH1 and GH3 β-glucosidases has argued that the deep and narrow substrate channel of GH1 β-glucosidases restricts glucose access to the active site and facilitates transglycosylation, thereby avoiding competitive inhibition while resulting in tolerance and stimulation^25^. However, why substrates containing glucose units are not excluded by the deep and narrow channel was not explained. In addition, the GH1 β-glucosidases intolerant to glucose were not accounted for in this study. Thus, whether the mechanism applies to all GH1 β-glucosidases is doubtful. There must be other factors than accessibility that contribute to the observed glucose effects on GH1 β-glucosidases.

Our laboratory has previously reported the cloning and characterization of two GH1 β-glucosidases, Bgl1A and Bgl1B, obtained from a marine microbial metagenome from the South China Sea by functional screening^19^,^26^. Bgl1A displayed high glucose tolerance, and was stimulated by low concentrations of glucose^19^. In contrast, the activity of Bgl1B was persistently inhibited by glucose^26^. Thus, the two β-glucosidases can be used as good representatives to investigate the glucose dependence of GH1 β-glucosidases. In the present study, using structural modeling and measurments of enzyme activities, combined with site-directed mutagenesis, several crucial sites and a potential mechanism for the glucose dependence of Bgl1A and Bgl1B were revealed. The mechanism may be extended to other GH1 β-glucosidases and consequently offer a starting point for the rational design of β-glucosidases aimed at optimizing the industrial application of these important enzymes by improving their glucose tolerance and stimulation effects.

## Results

### Structural modeling and comparison of Bgl1A and Bgl1B revealed a difference around the substrate channel

**Figure 1:**
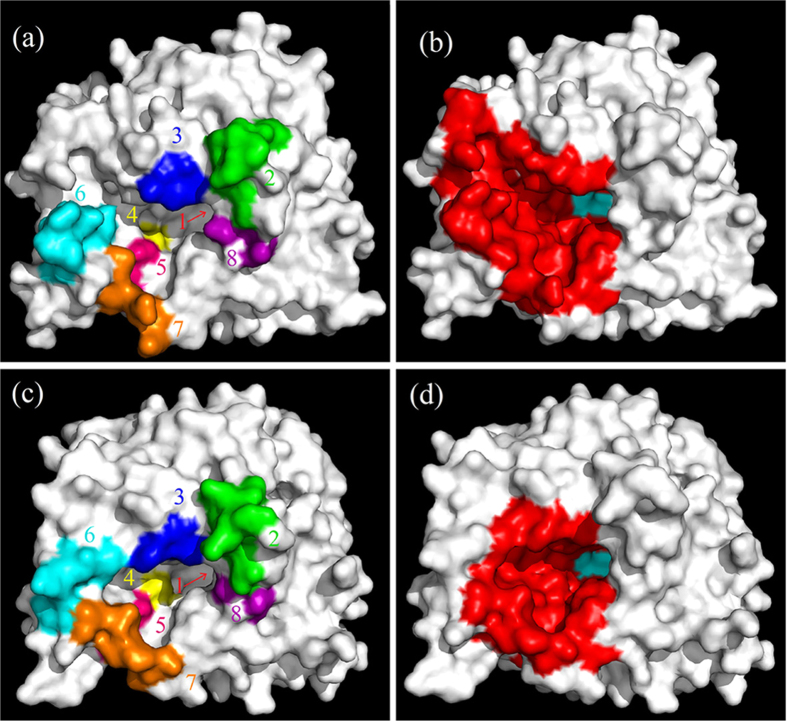
Surface representation of the structure models of Bgl1A (a,b) and Bgl1B (c,d). The sites colored and labeled with numbers are those different in sequence and located around the substrate channel (whose walls are colored in red in **b** and **d**), which penetrating from the structure surface into the inner and where the substrate approaches the active site (colored in light blue in **b** and **d**). The colors and numbers correspond to those defined in Supplementary Table S1. The imagines were made with PyMOL (http://www.pymol.org/)^32^.

Through functional screening from a marine metagenome, we previously obtained two bacterial β-glucosidases, Bgl1A and Bgl1B, which share high (56%) sequence identity but display distinct glucose dependence^19^,^26^. To reveal the molecular basis of the distinction, we have tried to crystallize the two enzymes for structural analysis, but failed. Fortunately, in Protein Data Bank (PDB), some β-glucosidases sharing >40% sequence identity with Bgl1A and Bgl1B are available. Therefore, we simulated the structures of the two proteins with the SWISS-MODEL program^27^. The structures of Bgl1A and Bgl1B were modeled using β-glucosidase structures 2O9P (BglB from *Paenibacillus polymyxa*) and 4PTV (HoBGLA from *Halothermothrix orenii*) as templates, respectively. Both structural models adopted the classical TIM beta/alpha-barrel structure of hydrolase, with global similarity and some local differences (Fig. 1). The global quality estimation score GMQE of the models were 0.76 and 0.75 for Bgl1A and Bgl1B, respectively, indicating moderate qualities of the models. Eight sites of Bgl1A and Bgl1B, which are different in sequence (Fig. S1) and structurally located around the substrate channel (Fig. 1), were supposed to be potential glucose-dependence relevant sites and thus mutated for further investigation (see discussion).

### Differences between wild-type enzymes and mutants revealed crucial sites for glucose dependence

**Figure 2:**
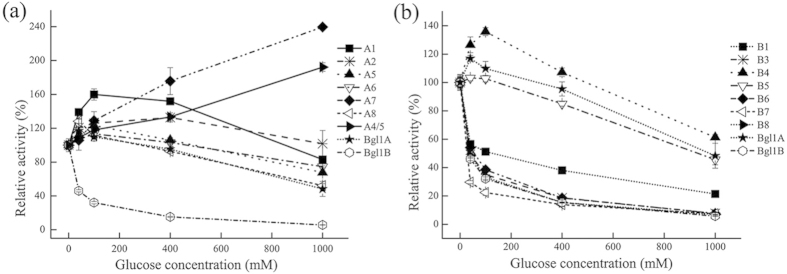
Effects of glucose on the relative activities of Bgl1A, Bgl1B and their interchange mutants. “An” stands for Bgl1A mutant with the site “n” mutated to its counterpart in Bgl1B; the same strategy was used to name Bgl1B mutants. “A4/5” stands for Bgl1A mutant with both site 4 and site 5 mutated.

To determine the relevance of the selected sites to the glucose dependence of Bgl1A and Bgl1B, mutants with residues at the chosen sites interchanged between the two proteins (Table S1) were constructed. The mutant proteins (named An for Bgl1A mutant with the site n mutated to its counterpart in Bgl1B; the same strategy was used to name Bgl1B mutants) were expressed, purified, and characterized. Except for A3, A4, and B2, whose activities were undetectable, all the mutants were tested. Compared with the wild-type enzyme, all the Bgl1A mutants remained glucose tolerant, with A7 being stimulated by glucose at all tested concentrations (Fig. 2a). This indicated that it is not a single site that determines the glucose tolerance of Bgl1A. In contrast, among the Bgl1B mutants, B4 (V_227_H_228_ mutated to F_227_T_228_) and B5 (N_301_V_302_ to Q_301_F_302_) exhibited substantial glucose tolerance, with B4 being significantly stimulated by low concentrations of glucose (Fig. 2b), distinct from the wild-type enzyme. This indicated that sites 4 and 5 synergistically account for the glucose intolerance of Bgl1B, consistent with observation that in Bgl1A, interchange mutations at any single one of the two sites could not eliminate the glucose tolerance. Presumably, interchange mutations at both 4 and 5 sites on Bgl1A should eliminate its glucose tolerance. However, mutant B4/5 (the residues 227-228 and 301-302 of Bgl1A mutated to those of Bg11B) was still glucose-tolerant and -stimulated (Fig. 2a). This implied that in addition to the local characteristics of some specific sites, other factors, such as the structural context, contribute to the glucose stimulation and tolerance of Bgl1A.

**Figure 3:**
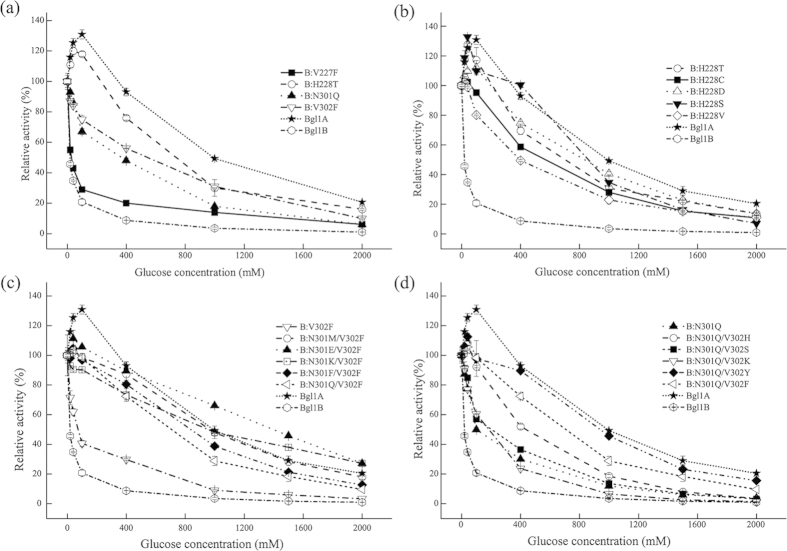
Effects of glucose on the relative activities of Bgl1A, Bgl1B and its mutants. (**a**) Bgl1A, Bgl1B and its single point interchange mutants at the sites 4 and 5. (**b**) Bgl1A, Bgl1B and its single point mutants of the 228th residue. (**c**) Bgl1A, Bgl1B and its double points mutants with the 301st residue varying while the 302nd maintained. (**d**) Bgl1A, Bgl1B and its double points mutants with the 302nd residue varying while the 301st maintained.

To further reveal the roles of the specific residues at sites 4 and 5 in the glucose dependence of Bgl1B, single-point interchange mutants of the two sites, namely, B:V227F B:H228T, B:N301Q, and B:V302F, were constructed. Among these mutants, only B:H228T exhibited glucose stimulation, essentially distinctive from wild-type Bgl1B, but similar to Bgl1A. In contrast, B:V227F displayed nearly the same glucose dependence as that of the wild-type enzyme (Fig. 3a). As for B:N301Q and B:V302F, although they exhibited weaker glucose inhibition, the enhanced tolerance was not as substantial as that of mutant B5, which involves both residues. These findings indicated that residue 228 is crucial for the contribution of site 4 to the glucose dependence of Bgl1B, whereas residues 301 and 302 should contribute synergically to site 5.

The crucial role of residue 228 in the glucose dependence of Bgl1B was further demonstrated by more single-point mutants at this site. The effect of mutation was residue-type dependent, with T, D, and S residues leading to initial stimulation, followed by enhanced tolerance, whereas C and V resulting in only alleviated inhibition (Fig. 3b). The difference between the effects of the mutations may result from the capability of the side chains of the residues to form hydrogen bonds because T, D, and S can provide hydrogen bond donor and acceptor, which were deficient in C and V.

The cooperation of the two residues at site 5 in Bgl1B was also confirmed by the mutants, in which one of the residues was replaced with its counterpart in Bgl1A and the other mutated to various residues. When residue 302 was maintained as F, all mutations at 301 resulted in substantial tolerance and slight stimulation (Fig. 3c). When residue 301 was maintained as Q, mutations to F, Y, and H at 302 led to substantial glucose tolerance and slight stimulation, whereas S and K at 302 resulted in only weaker inhibition (Fig. 3d). These observations implied that residue 302 contributes more to the glucose dependence of Bgl1B. It is postulated that the observed effects of the mutations are attributed to the large size of 301 side chain and the ring of 302, which may be beneficial to the interaction with glucose.

To reveal the mechanisms by which residues 228, 301, and 302 regulate the glucose dependence, the kinetic parameters of the wild-type enzymes and mutants were calculated at different glucose concentrations by using Michaelis-Menten equation (Fig. S2). For B:H228T and B:N301Q/V302F, the *V*_max_ increased initially but decreased as glucose concentration was further increased. Similar behavior was observed for Bgl1A (Table 1). In comparison, when glucose concentration was increased, the *V*_max_ of wild-type Bgl1B persistently decreased. While, for all the enzymes, the apparent *K*_m_ increased with glucose. These results indicated that glucose may inhibit the activity of Bgl1B by reducing substrate accessibility to the enzyme’s active site via competitive binding to the active site. For the Bgl1A and Bgl1B mutants, glucose may bind to other sites, probably the residues 228 and 301–302, in addition to the active site, and the glucose bound to these sites may stimulate the enzymatic activity through some mechanisms other than competitive inhibition.

### Docking of the ligands on the enzymes suggested several binding sites with different preferences

**Figure 4:**
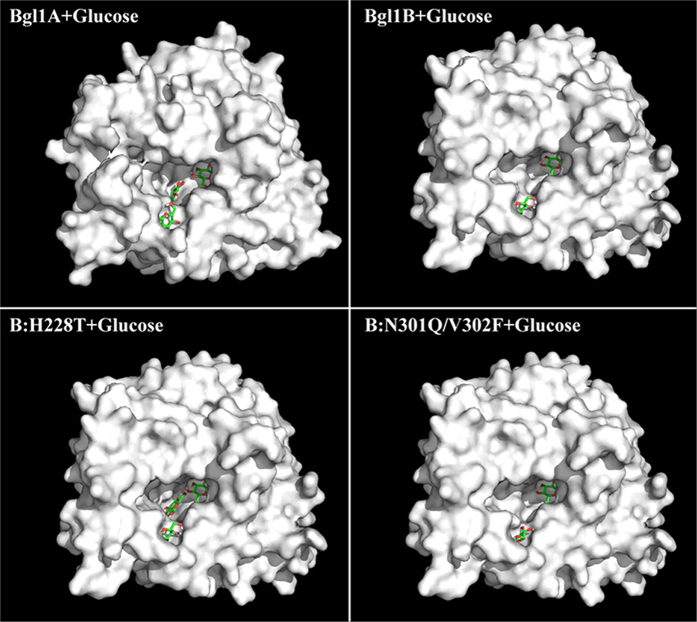
Glucose binding sites of Bgl1A, Bgl1B, B:H228T and B:N301Q/V302F determined by computational docking. In different proteins, most glucose bound to the substrate channel wall at a few same sites, around the channel bottom, middle and entrance, respectively. The binding were simulated by molecular docking and the images were made with PyMOL (http://www.pymol.org/)^32^, with glucose shown with colored sticks.

**Figure 5:**
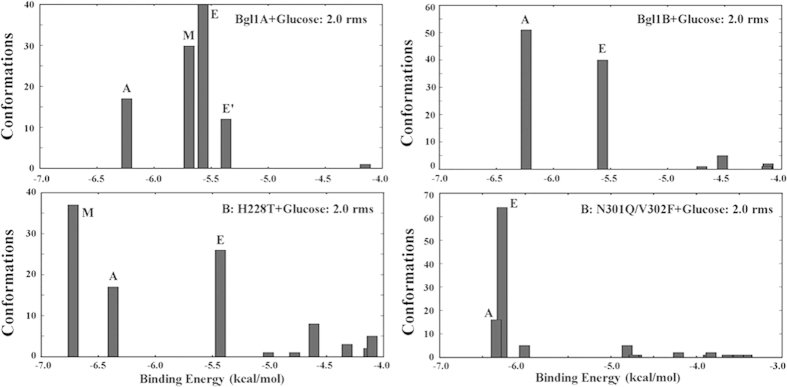
Binding energies and populations of the glucose bound to different sites in Bgl1A, Bgl1B, B:H228T and B:N301Q/V302F. The values were determined by molecular docking. “A”, “M” and “E” signify the sites glucose bound to, with “A” standing for the active site, “M” for the channel middle, and “E” for the channel entrance. “E′” stands for the channel entrance site bound with glucose adopting a different conformation from “E”. 2 rms means the root mean square between the conformations within a cluster is less than 2 Å.

To explore the sites and mechanism by which glucose affects the activities of β-glucosidases, interactions between the ligand and the enzymes were simulated by molecular docking. In Bgl1A, in addition to the active site at the bottom of the substrate channel, several other glucose binding sites were identified along the channel. Glucose bound to two of these sites, around residues 228 and 301–302 and located at the middle and entrance of the channel respectively, populated more, in other words with higher preference, than that bound to the bottom of the active site. In Bgl1B, the majority of glucose bound to the entrance site and the bottom of the active site, with higher preference to the latter. In B:H228T, most glucose bound with higher preference to the entrance and middle sites than to the bottom of the active site, as in Bgl1A. In B:N301Q/V302F, most glucose bound to the entrance and the bottom of the channel, as in wild-type Bgl1B, but with inverse preference (Figs 4 and 5). Notably, not all most preferred binding sites were those with the lowest energy or the highest accessibility (at the channel entrance). These results implied that it might be the glucose binding preference, more than the accessibility of the binding sites, that determines the glucose dependence of the β-glucosidases.

The relationship between the glucose binding preference and the glucose dependence of the β-glucosidases was supported by the docking data of other mutants. All mutants with enhanced glucose tolerance displayed glucose binding preference to sites other than the active site (Table S2). Most of the Bgl1A mutants with significant glucose stimulation, i.e. A1, A2, and A7, displayed higher preference at the middle site (Table S2). However, A5 and A4/5, which lost the local structural characteristics of Bgl1A at site 5 (entrance of the channel), did not lose glucose binding to site 5 as expected. In addition, both of them, as well as A8, did not bind glucose at site 4 (middle of the channel), while all of them displayed more or less glucose stimulation (Fig. 2a). For the Bgl1B mutants, only B4, B:H228S, and B:H228T showed preference at the middle site (Table S2), which is consistent with their unique glucose stimulation property (Fig. 3b). B:H227F, like Bgl1B, showed relatively higher preference at the active site. The similarity between their glucose binding preferences is consistent with the similarity between their glucose inhibition profiles.

Molecular docking illustrated the cause of lower interaction energies between glucose and the crucial residues in Bgl1A and the Bgl1B mutants compared with wild-type Bgl1B. The hydroxyl proton of the side chain of T228 in Bgl1A and B:H228T can form hydrogen bonds with glucose (Fig. S3), which is consistent with the previous speculation for the glucose dependence of residue 228 relevant mutants: T, D, and S can offer atoms as hydrogen bond donor and acceptor, which were absent in C and V, thus leading to distinct glucose dependence. Moreover, the large size of H residue in Bgl1B may result in steric hindrance for glucose binding (Fig. 4). For residues 301 and 302, compared with N and V in Bgl1B, Q and F in Bgl1A and B:N301Q/V302F may offer more hydrogen bonding because of the extended side chain and stronger hydrophobic interaction between the aromatic ring and saccharide, respectively (Fig. S3).

Compared with glucose, the substrate p-nitrophenyl-β-D-glucopyranoside (pNPG) can bind to more sites along the channel, some of which overlap glucose-binding sites. Among the putative pNPG binding sites, the active site at the bottom of the substrate channel always exhibited the lowest energy (Fig. S4), which may be attributed to the additional pNP group that can provide larger interacting surface and thus contribute to higher affinity.

**Figure 6:**
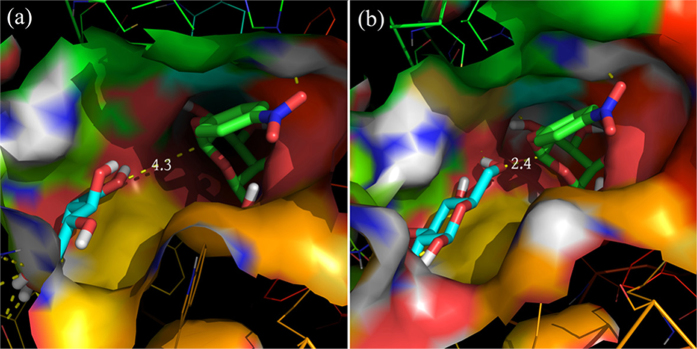
Geometry of glucose and pNPG docked on (a) Bgl1A and (b) B:H228T. Glucose and pNPG are shown with thick sticks, with oxygen and hydrogen colored in red and white respectively, carbon colored in cyan and green in glucose and pNPG respectively, and nitrogen colored in blue in pNPG. The lines link the oxygen atom of 6’ −OH of glucose and the carbon atom of the β-glycosidic bond of pNPG, with the distance (in unit of Å) labeled beside. The images were made with PyMOL (http://www.pymol.org/)^32^.

Notably, when glucose bound to the middle sites of substrate channel in Bgl1A and B:H228T, its 4’- and 6’-OH group were adjacent to the glycosidic bond of pNPG bound to the active site (Fig. 6). This geometry will be advantageous for glucose to act as a nucleophile in place of water, thereby resulting in transglycosylation reactions rather than hydrolysis.

### Transglycosylation activity of enzymes offered potential explanation for glucose stimulation

**Figure 7:**
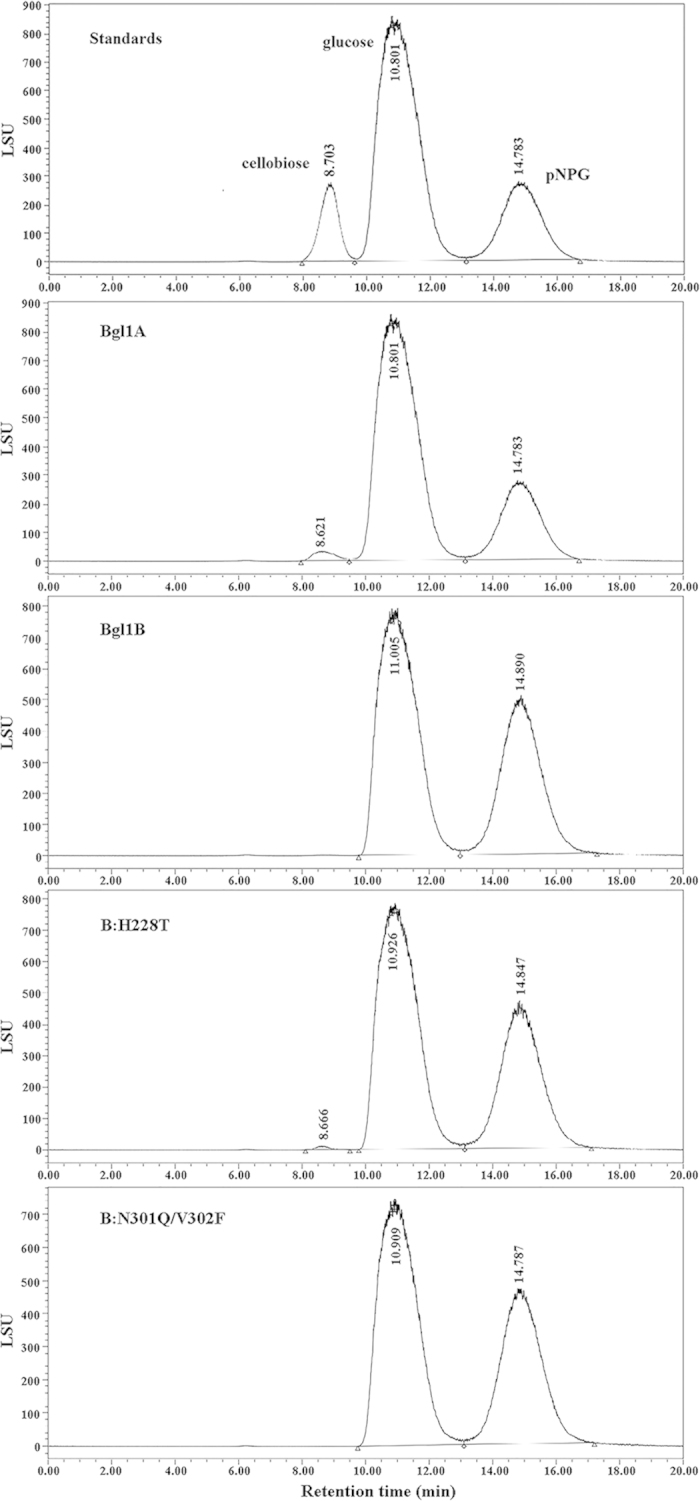
HPLC analysis for the products of Bgl1A, Bgl1B, B:H228T and B:N301Q/V302F acting on pNPG. The standard chromatogram for cellobiose, glucose and pNPG is shown in the top panel for reference.

As mentioned previously, many studies attributed the glucose stimulation of β-glucosidases to their transglycosylation activity^18^, which may be the case for Bgl1A and Bgl1B mutant H228T as supported by the docking results. To confirm this assumption, the products of the enzymes acting on pNPG were analyzed with high performance liquid chromatography (HPLC). The results showed that additional peaks were observed besides those for the substrate pNPG and the product glucose (Fig. 7). The elution times of the additional peaks were consistent with that of cellobiose, indicating a disaccharide produced by transglycosylation activity.

## Disscussion

As the sequence identities between Bgl1A and Bgl1B and the PDB templates are not very high, limited global qualities (with moderate GMQE about 0.75, and relatively low local composite scoring function QMEAN4 about -5.3) of the models are expected. However, the reliabilities of the simulated results based on these models and derived conclusions are considerably valid, as the local qualities of the models at the examined sites (228, 301 and 302) are rather high (with the reliability scores, calculated from the QMEAN values, being about 0.81, 0.66 and 0.51 respectively). More critical, the results of biochemical characterization and site-directed mutagenesis fit with those of structure simulation very well, supporting the validity of the models.

The modeled structures of Bgl1A and Bgl1B exhibit substrate channels of similar depth and width, supporting the idea that there are other mechanisms than channel width and depth that determine their different glucose dependence. Glucose products can inhibit the enzymatic activity of β-glucosidases by competing with substrate to bind directly to the active site. Presumably, glucose could affect active site indirectly via factors related to substrate binding, such as perturbing the water matrix and steric geometry in the substrate channel. Hence, glucose tolerance and stimulation may result from glucose binding to other sites, probably at the entrance or middle of the substrate channel. Therefore, 8 sites were chosen to perform mutation to further investigate the effects of glucose on β-glucosidases.

Based on biochemical characterization and ligand docking of the wild-type enzymes and mutants, a mechanism for the effects of glucose on the activity of GH1 β-glucosidases was proposed. This mechanism emphasizes the following interactions between glucose and GH1 β-glucosidases: (1) Besides binding to the active site, glucose, as well as the substrate, can bind to other sites (part of which are identical for both glucose and the substrate) along the substrate channel with varying affinities. (2) The response of GH1 β-glucosidases to glucose depends on relative affinity/preference of glucose binding to different sites, being inhibition when glucose preferentially binds to the active site and tolerance when it prefers to bind to other sites. (3) Glucose stimulation results from the geometry of certain binding sites, where the bound glucose will enhance substrate cleavage activity through transglycosylation or other mechanisms such as allosteric effects.

**Figure 8:**
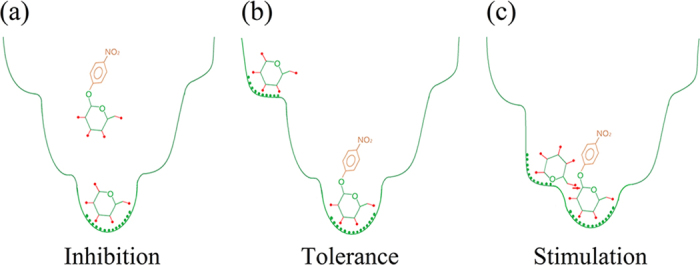
Schematic illustration of the proposed mechanism for glucose effects on Bgl1B (a), Bgl1A (b,c) and their mutants. The outside profile denotes the substrate channel wall. The arrow points to the anomeric carbon atom which is attacked by glucose in transglycosylation. The dots on the channel wall signify the interactions between the enzyme and the ligands. The red sticks with red dots at termini denote bonds between carbon and hydroxyl group.

This mechanism perfectly accounts for the results observed in this study. In Bgl1B, glucose prefers to bind to the active site at the bottom of substrate channel, thus affecting the enzyme activity via competitive inhibition (Fig. 8a). In Bgl1A, Bgl1B mutants H228T and N301Q/V302F, glucose preferentially binds to outside sites at the middle and entrance of the channel, where the bound glucose does not hinder substrate binding to the active site, therefore leading to glucose tolerance (Fig. 8b,c). Moreover, glucose bound to the middle of the channel can stimulate substrate cleavage activity by transglycosylation because of close proximity of this location to substrate (Fig. 8c), this is reminiscent to the case of another GH1 β-glucosidase, rice BGlu1^28^.

Notably, all the Bgl1A mutants constructed in this study, including both single and double sites, showed slight glucose stimulation in addition to tolerance. This observation can be explained by indirect factors influencing enzyme-ligand interaction. In addition to some specific crucial sites, such as residues 228, 301, and 302, the overall structural features also contribute to glucose’s preferential binding to sites other than the active site. This is in good agreement with our previous study that showed the indispensable role of residue 184 in Bgl1B, which is also conserved in Bgl1A^29^. Moreover, in addition to binding to the middle site to enhance the cleavage activity through transglycosylation, glucose may also bind to other sites to stimulate enzyme activity through some unknown mechanisms. The details of the overall structural features and other mechanisms contributing to glucose stimulation are to be elucidated.

Our results demonstrated the previous arguments that in GH1 β-glucosidases there are sites other than the active site that can bind glucose^15^,^23^ and glucose may promote cleavage activity by transglycosylation^17^,^18^. In addition, our study extended the results from a recent report that attributed the glucose tolerance to the accessibility of the active site to glucose^25^. As shown in the present study, not only the accessibility but also the binding energy, both of which were related to the structure, determined the population of the bound glucose. Thus, relative affinity/preference rather than accessibility of the glucose binding sites is crucial for the glucose dependence of GH1 β-glucosidases.

Our mechanism may be extended to other GH1 β-glucosidases, with some issues modified by specific circumstances. First, the number and location of the binding sites vary for different proteins. Second, relative affinity/preference of the binding sites varies from protein to protein. Both number/location and relative affinity/preference of the binding sites depend on structural characteristics and determine the variation of the glucose dependence of GH1 β-glucosidases. For example, in HiGB, deep and narrow substrate channel hinders glucose access to the active site, consequently leading to its preferential binding to an outside site, F348, which is suitable for transglycosylation, thus resulting in glucose stimulation and tolerance^25^. Molecular docking for HiGB showed that glucose actually bind to the active site as well, but with lower preference (data not shown). Another example is rice BGlu1, in which hexose was supposed to be positioned by Y131, hence ready for nucleophilic attack at anomeric carbon and leading to high transglycosylation activity and glucose stimulation^28^. These are essentially similar to the case of Bgl1A, except that the specific outside glucose-binding sites are different. Reasonably, in GH1 β-glucosidases that are intolerant to glucose, glucose might bind to the active site more favorably than to the outside sites, if any, thus resulting in competitive inhibition.

Despite substantial evidence, atom-resolution structures and molecular dynamics simulation are needed to further improve this mechanism, which should be useful for the design of catalytically enhanced GH1 β-glucosidases for industrial applications, such as biofuel production, in which glucose tolerance and stimulation are highly desirable. In addition, this mechanism may help our understanding of product effects on other enzymes especially hydrolases.

## Methods

### Materials

*Escherichia coli* strains BL21 (DE3) were obtained from TransGen Corp. (Beijing, PR China). PrimeSTAR HS DNA Polymerase, all restriction endonucleases, and T4 DNA ligase were purchased from TAKARA (Dalian, PR China). The expression vector pET22b(+) was purchased from Novagen (Madison, USA). *p*-nitrophenyl-β-D-glucopyranoside (pNPG) was purchased from Aladdin-Reagent (Shanghai, PR China). The Ni^2+^-NTA resin was purchased from Novagen Corp. (Darmstadt, Germany). All other chemicals and reagents were of analytical grade and purchased from commercial sources, unless otherwise stated.

### Homology modeling and sequence alignment

Currently, no crystal structures are available for wild-type Bgl1A and Bgl1B to evaluate the relationship between the glucose tolerance and structure of β-glucosidase. In the PDB database, the β-glucosidase BglB (2O9P)^30^ from *Paenibacillus polymyxa* shares 43% sequence identity with Bgl1A, and *Ho*BGLA (4PTV)^31^ from *Halothermothrix orenii* shares 42% sequence identity with Bgl1B. Thus, the 3D structure models of Bgl1A and Bgl1B, based on 2O9P and 4PTV, respectively, were constructed using the homology modeling by SWISS-MODEL. The structures were visualized using the visualization tool PyMOL (http://www.pymol.org/)^32^. Sequence alignment of Bgl1A and Bgl1B was performed by Clustal X^33^, and the result was formatted by ESPript version 3.0^34^.

### Site-directed mutagenesis

Construction of mutants based on SOE-PCR^35^ (splicing by overlap extension-polymerase chain reaction) was performed in two steps. First, the previously reported recombinant plasmids of pET-22b(+)-*bgl1a* and -*bgl1b*^19^,^26^ were used as templates for mutagenesis; mutagenic oligonucleotide primer pairs of wild-type forward (WF) and mutant site reverse (MR), as well as mutant site forward (MF) and wild-type reverse (WR) were used to amplify the upstream and downstream fragments, named as MU and MD, respectively. Subsequently, MU and MD fragments were assembled using WF and WR as primers to produce the complete DNA sequences of the mutants. All the primers are listed in Supplementary Table S3. All the PCR products were digested with *Nde*I and *Xho*I and ligated into pET-22b(+) vector, thereby generating the mutated plasmids with six histidine codons attached to the 3′ end of the inserted target sequences. The recombinant plasmids were validated by sequencing.

### Protein expression and purification

The recombinant pET22b(+) plasmids containing the β-glucosidase genes were transformed into BL21(DE3) cells. For the expression of the wild-type enzymes and mutants, cells were grown in LB medium supplemented with 100 μg mL^−1^ ampicillin at 37 °C to an OD_600_ of approximately 0.6 before being induced with 0.2 mM IPTG at 16 °C. Overnight cultures were harvested by centrifugation (5 min at 8000 rpm), and the pellet was resuspended in cold 20 mM Tris-HCl buffer (pH 7.9) containing 500 mM NaCl and 5 mM imidazole, disrupted by ultrasonic processor (Scientz, PR China). The suspension was centrifuged for 30 min at 4 °C and 12000 × *g* to remove the cell debris and intact cells. The supernatant was loaded on a 2 mL Ni^2+^-NTA affinity chromatography column. The column was washed with 10 column volumes of Tris-HCl containing 30 mM imidazole before the protein was eluted with Tris-HCl buffer containing 200 mM imidazole. After dialysis against 50 mM Na_2_HPO_4_-citric acid buffer (pH 7.0), the purified protein was concentrated by ultrafiltration and stored at 4 °C. All the proteins were expressed and purified as described above. Protein purity was assessed by SDS-PAGE (12% gel). The concentration of the protein sample was determined using BioPhotometer plus (Eppendorf, Germany).

### Enzyme assay and kinetics study

β-glucosidase activity was tested with pNPG as a substrate. The assay mixture consisted of 25 μL of protein stock sample and 475 μL of 50 mM Na_2_HPO_4_-citric acid buffer (pH 7.0) with 5 mM pNPG. Factors including enzyme concentration and incubation time were initially screened in the pre-experiments, so that experimental conditions employed in all experiments guarantee the estimation of initial velocities V_0_ (hydrolysis of no more than 5% initial substrate, linear response of product formation in respect to reaction time). The enzyme activity was estimated by measuring the optical absorption at 405 nm by pNP in the product. Reactions with heat-treated samples were used as controls. The kinetic parameters of the β-glucosidases were tested under optimal conditions, and *K*_m_ and *V*_max_ were calculated by nonlinear regression of the Michaelis-Menten equation (equation (1)) with OriginLab 8.5 (OriginLab Corporation, USA). All measurements were performed in triplicate and repeated at least twice.




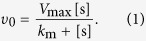




The effect of glucose on the β-glucosidases was evaluated by measuring the activity at different glucose concentrations, ranging from 0.0 M to 2.0 M. The relative activity was defined as the relative value to the activity of the control without glucose.

All the experiments data were an average of three parallel replicates, and the error bars in the figures were standard deviations.

### Protein-ligand docking

AutoDock 4.0^36^ was employed for docking to discover the potential glucose binding sites in the substrate channel. The 3D structures of the proteins were generated by SWISS-MODEL, those of glucose and pNPG, both in chair conformation, were downloaded from PDB as a part of 2O9T (a β-glucosidase from *Bacillus polymyxa* with glucose) and 3AI0 (a β-glucosidase from termite *Neotermes koshunensis* with pNPG), respectively. The files were transformed to .pdb form by PyMOL, Gasteiger charges and essential hydrogen atoms were added using the AutoDock tools. The rotatable bonds (carbon-oxygen, carbon-nitrogen, exocyclic carbon-carbon, and glucosidic bonds) in the ligand were assigned with AutoDock tools, and the ligand docking was performed with the AutoDock Lamarckian Genetic Algorithm (LGA, runs 100). The receptors were enclosed in a grid with 0.375 Å spacing, and the ligands were allowed to move within the whole substrate channel to achieve the lowest energy conformation. Different energy terms, including intermol energy (vdm + hbond + desolv energy + electrostatic energy), internal energy, torsional energy, and binding energy, can be obtained from AutoDock and estimated G binding = intermol energy + torsional energy. The lowest energy (in vacuum) conformation was employed for further analysis.

### Transglycosylation activity assay and HPLC analysis

Transglycosylation activity was determined by probing the production of disaccharide using pNPG and glucose as the glycosyl donor and acceptor, respectively. A 1 mL reaction mixture containing 0.2 U of enzyme, 100 mM glucose, and 5 mM pNPG in a 50 mM Na_2_HPO_4_-citric acid buffer (pH 7.0) was incubated at 35 °C. Samples of 20 μL were withdrawn at different time intervals (5 min to 2 h) and applied to a HPLC system (Waters, USA) to detect the transglycosylation product. The HPLC system was equipped with a Carbomix Ca-NP (7.8 mm × 300 mm) column (Sepax Technologies Inc., USA) and an evaporative light-scattering detector 2424 (Waters, USA) with an evaporator temperature at 70 °C. The eluting solution was 20:80 (v:v) of acetonitril:water.

## Additional Information

**How to cite this article**: Yang, Y. *et al.* A mechanism of glucose tolerance and stimulation of GH1 β-glucosidases. *Sci. Rep.* **5**, 17296; doi: 10.1038/srep17296 (2015).

## Supplementary Material

Supplementary Information

## Figures and Tables

**Table 1:** Kinetic parameters for Bgl1A, Bgl1B and its interchange mutants.

Enzyme	Glucose (mM)	*K*_m (mM)_	*V*_max (μM min^−1^)_	*k*_cat*/*_ *K*_m (s^−1^ mM^−1^)_
Bgl1A	0	0.69 ± 0.07	2.70 ± 0.08	35.51
	50	0.84 ± 0.04	3.34 ± 0.05	36.08
	100	1.13 ± 0.06	3.87 ± 0.07	31.08
	400	2.08 ± 0.16	2.82 ± 0.08	12.30
Bgl1B	0	0.27 ± 0.02	12.58 ± 0.25	74.72
	50	0.70 ± 0.04	4.03 ± 0.07	9.23
	100	1.07 ± 0.05	2.72 ± 0.04	4.08
B:H228T	0	0.36 ± 0.03	10.14 ± 0.16	24.18
	50	2.00 ± 0.10	15.01 ± 0.29	6.44
	100	2.24 ± 0.19	14.10 ± 0.47	5.40
	400	4.52 ± 0.45	8.87 ± 0.44	1.68
B:N301Q/V302F	0	0.83 ± 0.07	8.84 ± 0.23	2.86
	50	1.14 ± 0.07	8.83 ± 0.17	2.08
	100	2.98 ± 0.18	11.20 ± 0.290	1.01
	400	5.30 ± 0.40	9.00 ± 0.396	0.46
