# Supplementary material for: A mechanism of glucose tolerance and stimulation of GH1 β-glucosidases
Source: Sci Rep. 2015 Nov 25;5:17296. doi: 10.1038/srep17296 (PMC4658561; doi:10.1038/srep17296)
Supplement: Supplementary Information [file srep17296-s1.pdf]

A mechanism of glucose tolerance and stimulation of GH1 β-glucosidases

Yang Yang[1,2,+], Xinxin Zhang[1,2,+], Qiang Yin[1,2], Wei Fang[1,2], Zemin Fang[1,2], Xiaotang Wang[3], Xuecheng Zhang[1,2,*] and Yazhong Xiao[1,2,*]

[1] School of Life Sciences, Anhui University, Hefei, Anhui 230601, China

[2] Anhui Provincial Engineering Technology Research Center of Microorganisms and Biocatalysis, Hefei, Anhui 230601, China

[3] Department of Chemistry & Biochemistry, Florida International University, Miami, Florida 33199, United States

[+] these authors contribute equally to this work

[*] corresponding authors, emails: turenzh@ahu.edu.cn and yzxiao@ahu.edu.cn

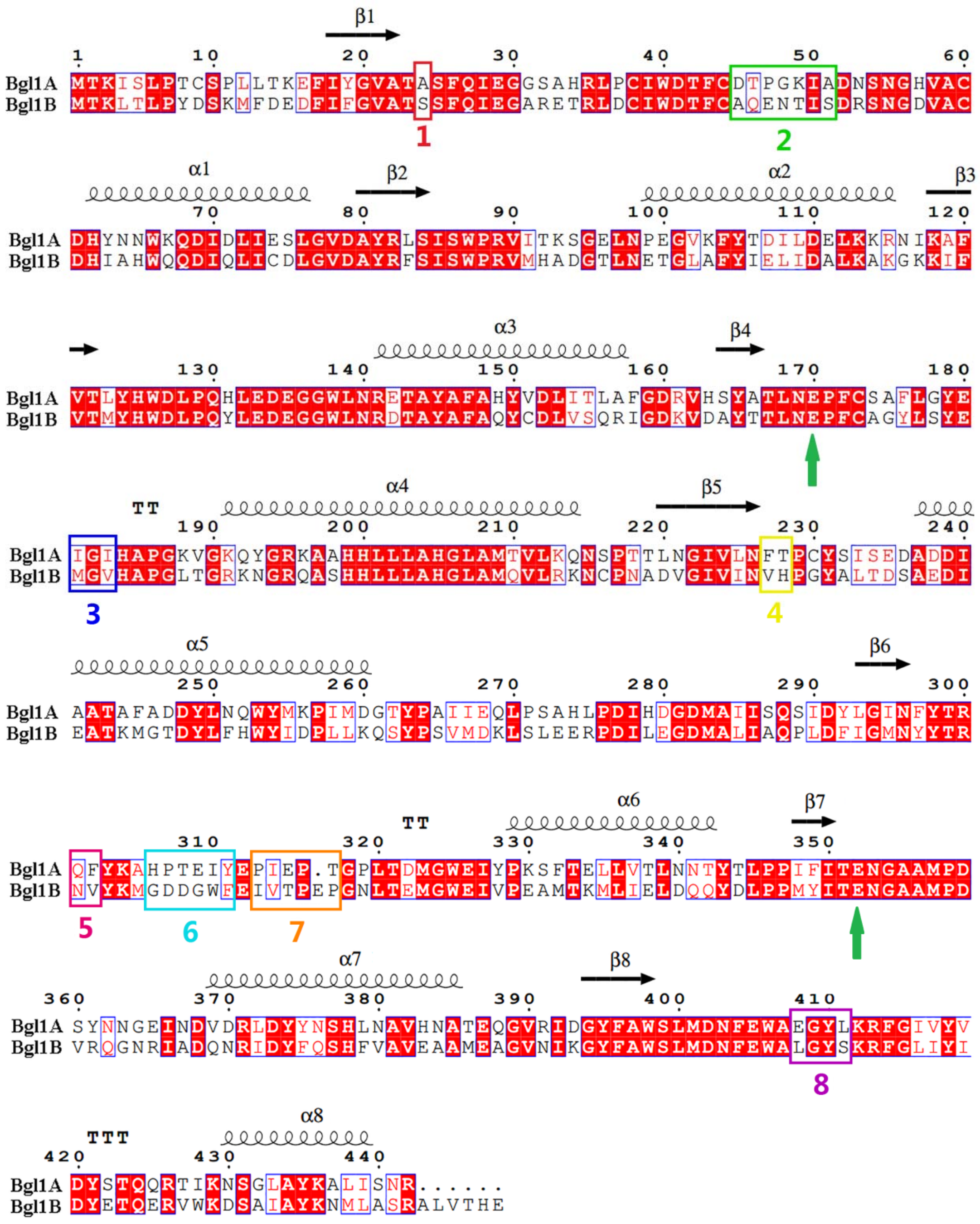

Figure S1. Sequence alignment for Bgl1A and Bgl1B. Identical residues are highlighted in red color, and similar ones are in hollow frames. Predicted secondary structures are denoted above the sequence, with coil standing for α helix, arrow for β strand, and "T" for turn. The sequences in colored frames and labeled with numbers are those different in sequence and located along the substrate channel. The colors and

numbers are consistent with those in Fig. 1 and Fig. 2

Table S1. List of interchange mutants of Bgl1A and Bgl1B at selected glucose-dependence relevant sites.

Mutant No.	Sequence number in Bgl1A (Bgl1B)	Amino acid(s) in Bgl1A		Amino acid(s) in Bgl1B
1	24	A	⟷	S
2	45-51	DTPGKIA	⟷	AQENTIS
3	181-183	IGI	⟷	MGV
4	227-228	FT	⟷	VH
5	301-302	QF	⟷	NV
6	305-311	AHPTEIY	⟷	MGDDGWF
7	313-317(313-318)	PIEPT	⟷	IVTPEP
8	408-411(409-412)	EGYL	⟷	LGYS

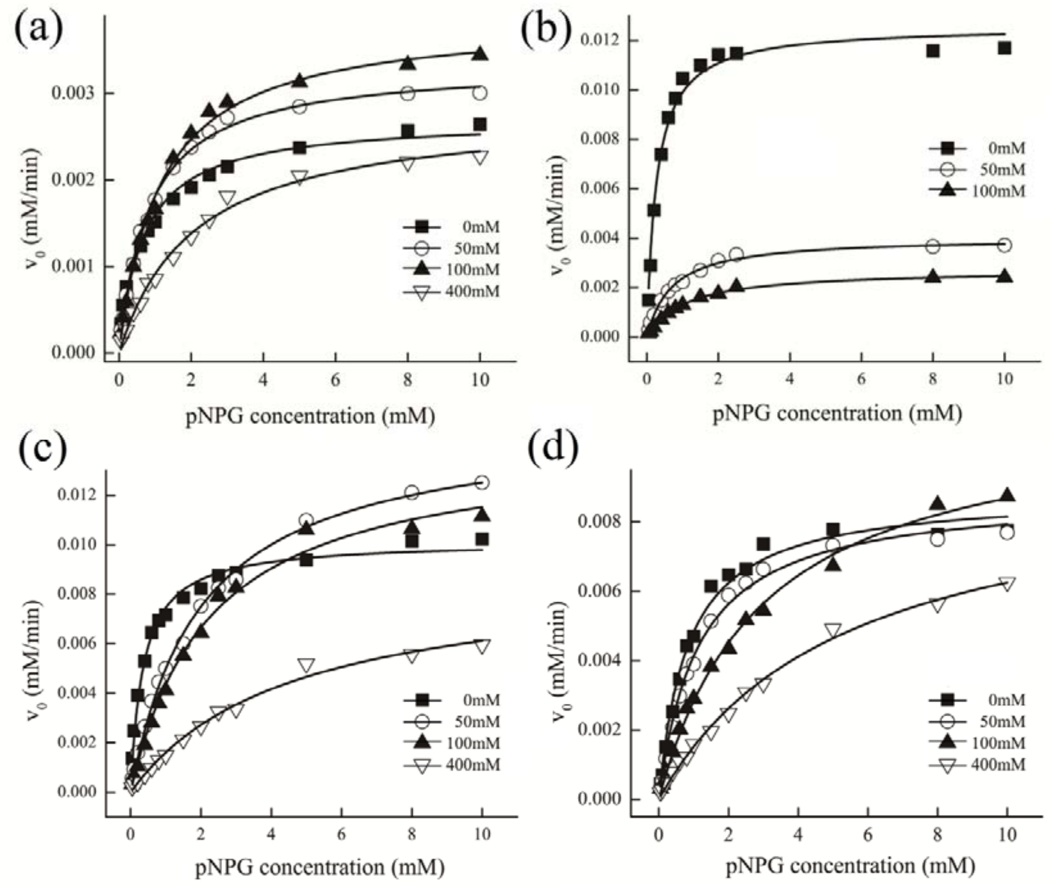

Figure S2. Enzyme kinetics of (a) Bgl1A, (b) Bgl1B, (c) B:H228T, and (d) B:N301Q/V302F in different concentrations of glucose. The symbols are tested values, and the lines are fitted curves calculated by the Michaelis-Menten equation.

Table S2. Populations of the glucose bound to different sites in Bgl1A, Bgl1B and their mutants. The populations were determined by molecular docking. "A", "M" and "E" signify the sites glucose bound to, with "A" standing for the active site, "M" for the channel middle, "E" for the channel entrance, and "other" for other sites.

Mutants	A	M	E	other
Bgl1A	17	30	52	1
A1	8	23	68	1

A2	13	24	63	0
A4/5	7	0	90	3
A5	17	0	80	3
A6	12	16	62	10
A7	10	31	33	26
A8	10	0	68	22
Bgl1B	51	0	40	9
B:V227F	38	0	54	8
B:H228C	25	2	72	1
B:H228D	25	0	74	1
B:H228S	17	33	50	0
B:H228V	18	0	76	6
B4(B:V227F/H228T)	12	36	45	7
B:V302F	20	0	78	2
B:N301M/V302F	12	0	84	4
B:N301E/V302F	12	0	86	2
B:N301K/V302F	13	0	82	5
B:N301F/V302F	13	0	87	0
B5(B:N301Q/V302F)	21	0	73	6
B:N301Q	11	0	77	12
B:N301Q/V302H	15	0	75	10
B:N301Q/V302S	9	0	83	8
B:N301Q/V302K	13	0	82	5
B:N301Q/V302Y	12	0	86	2

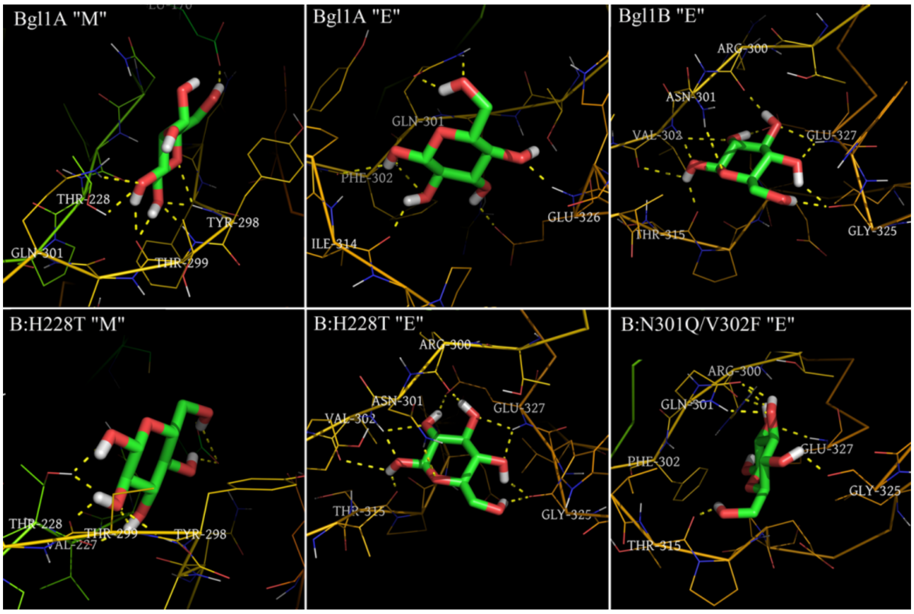

Figure S3. Interactions between docked glucose and Bgl1A, Bgl1B, B:H228T and B:N301Q/V302F. Glucose is shown with thick sticks, with carbon atoms colored in green, oxygen in red and hydrogen in white. Proteins are shown with thin sticks, with carbon atoms colored in yellow, oxygen in red, nitrogen in blue and hydrogen in white. Hydrogen bonds are signified by dotted lines. “M” and “E” denote the channel middle site and the channel entrance site, respectively.

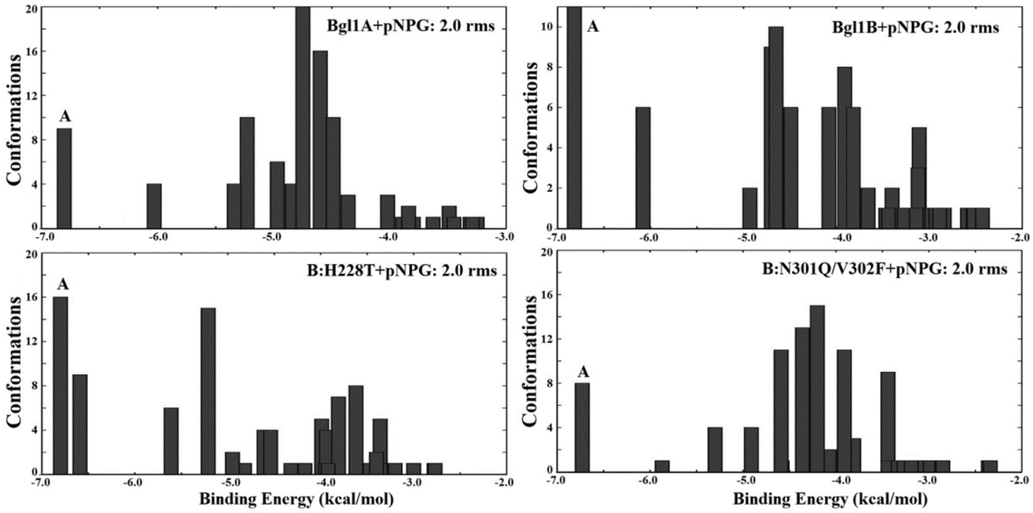

Figure S4. Binding energies and populations of pNPG bound to different sites in

Bgl1A, Bgl1B, B:H228T and B:N301Q/V302F. The values were determined by molecular docking. “A” stands for the active site. 2 rms means the root mean square between the conformations within a cluster is less than 2 Å.

Table S3. Sequences of the primers used for site-directed mutation of *Bgl1A* and *Bgl1B*.

Primer	Sequence 5’→3’
Bgl1A-F	GGAATTCCATATGACTAAAATATCTTTACCAAC
Bgl1A-R	CCGCTCGAGTCTATTTGAGATTAATGCTT
Bgl1A-F1	GTAGCAACATCATCTTTCCAAATAGAAGG
Bgl1A-R1	GGAAAGATGATGTTGCTACACCAT
Bgl1A-F2	GCTCAAGAAAACACTATTTCTGATAACTCAAATGGGC
Bgl1A-R2	AGAAATAGTGTTTTCTTGAGCACAAAAGGTATCCCAG
Bgl1A-F3	ACGAAATGGGGGTGCATGCGCCAGGGAAAGT
Bgl1A-R3	CGCATGCACCCCCATTTCGTAACCTAAAAAAGCACT
Bgl1A-F4	GCTTAACGTCCACCCCTGTTATAGCATC
Bgl1A-R4	CAGGGGTGGACGTTAAGCACGATACCGT
Bgl1A-F5	ATACCCGTAACGTTTATAAAGCGCACCCTACT
Bgl1A-R5	CTTTATAAACGTTACGGGTATAAAAGTT
Bgl1A-F6	ATGGGGGATGACGGTTGGTTCGAGCCAATAGAGCCT
Bgl1A-R6	GAACCAACCGTCATCCCCCATTTTATAAAATTGACG
Bgl1A-F7	ATAGTCACGCCTGAACCTGGCCCGCTAACCGATATG
Bgl1A-R7	AGGTTCAGGCGTGACTATCTCATATATTTCAGTAG
Bgl1A-F8	CACTAGGTTATAGTAAAAGATTTGGTATAG
Bgl1A-R8	CTTTTACTATAACCTAGTGCCCATTCAAAGT
Bgl1B-F	GGAATTCCATATGACAAAATTAACTTTACCT
Bgl1B-R	CCGCTCGAGTTCGTGGGTAACAAGTG

Bgl1B-F1	GTCGCCACCGCGTCATTCCAAATTGAAGG
Bgl1B-R1	GAATGACGCGGTGGCGACACCAAAGAT
Bgl1B-F2	GATACTCCAGGTAAAATAGCTGATCGTTCCAACGGGG
Bgl1B-R2	AGCTATTTTACCTGGAGTATCACAGAACGTATCCCAAAT AC
Bgl1B-F3	ATGAAATTGGCATTCATGCGCCAGGTTTAAC
Bgl1B-R3	CGCATGAATGCCAATTTCATAACTTAAATAAC
Bgl1B-F4	GATAAATTTTACTCCTGGTTATGCATTAAC
Bgl1B-R4	CCAGGAGTAAAATTTATCACGATCCCGACATCGG
Bgl1B-F5	ACGCGTCAATTTTATAAGATGGGGGATGAC
Bgl1B-R5	CTTATAAAATTGACGCGTGTAGTAATTCATAC
Bgl1B-F6	GCGCACCCTACTGAAATATATGAAATAGTCACGCCTG
Bgl1B-R6	ATATATTTCAGTAGGGTGCGCCTTATAAACGTTACGCG
Bgl1B-F7	GAACCAATAGAGCCTACTGGTAATTTAACAGAAATG
Bgl1B-R7	ACCAGTAGGCTCTATTGGTTCGAACCAACCGTCATC
Bgl1B-F8	GGCAGAAGGTTACTTAAAGCGCTTTGGTTTGAT
Bgl1B-R8	CGCTTTAAGTAACCTTCTGCCCACTCAAAATTATC
B:V227F-F	ATCGTGATAAATTTTCACCCTG
B:V227F-R	CAGGGTGAAAATTTATCACGAT
B:H228T-F	GATAAATGTCACTCCTGGTTATGC
B:H228T-R	GCATAACCAGGAGTGACATTTATC
B:N301Q-F	ACACGCGTCAAGTTTATAAGATGG
B:N301Q-R	CCATCTTATAAACTTGACGCGTGT
B:V302F-F	ACACGCGTAACTTTTATAAGATGG
B:V302F-R	CCATCTTATAAAAGTTACGCGTGT
B:H228C-F	TAAATGTCTGTCCTGGTTATGC
B:H228C-R	GCATAACCAGGACAGACATTTA
B:H228D-F	TAAATGTCGATCCTGGTTATGC
B:H228D-R	GCATAACCAGGATCGACATTTA

B:H228S-F	TAAATGTCTCACCTGGTTATGC
B:H228S-R	GCATAACCAGGTGAGACATTTA
B:H228V-F	TAAATGTCGTCCCTGGTTATGC
B:H228V-R	GCATAACCAGGGACGACATTTA
B:N301M/V302F-F	ACACGCGTATGTTTTATAAGATGG
B:N301M/V302F-R	CCATCTTATAAAACATACGCGTGT
B:N301E/V302F-F	ACACGCGTGAATTTTATAAGATGG
B:N301E/V302F-R	CCATCTTATAAAATTCACGCGTGT
B:N301K/V302F-F	ACACGCGTAAGTTTTATAAGATGG
B:N301K/V302F-R	CCATCTTATAAAACTTACGCGTGT
B:N301F/V302F-F	ACACGCGTTTCTTTTATAAGATGG
B:N301F/V302F-R	CCATCTTATAAAAGAAACGCGTGT
B:N301Q/V302F-F	ACACGCGTCAATTTTATAAGATGG
B:N301Q/V302F-R	CCATCTTATAAAATTGACGCGTGT
B:N301Q/V302H-F	ACACGCGTCAACACTATAAGATGG
B:N301Q/V302H-R	CCATCTTATAGTGTTGACGCGTGT
B:N301Q/V302S-F	ACACGCGTCAATCATATAAGATGG
B:N301Q/V302S-R	CCATCTTATATGATTGACGCGTGT
B:N301Q/V302K-F	ACACGCGTCAAAAGTATAAGATGG
B:N301Q/V302K-R	CCATCTTATACTTTTGACGCGTGT
B:N301Q/V302Y-F	ACACGCGTCAATATTATAAGATGG
B:N301Q/V302Y-R	CCATCTTATAATATTGACGCGTGT
